# Home-Based Care in the Context of COVID-19: Disruptions, Innovations, and Lessons for the Future

**DOI:** 10.1093/geroni/igab046.2050

**Published:** 2021-12-17

**Authors:** Emily Franzosa, Katherine Ornstein

## Abstract

During the initial surge of the COVID-19 pandemic, home-based primary and palliative care (HBPC) practices played a valuable role in maintaining access to health-related services and keeping older, medically complex patients out of hospitals and congregate settings that could heighten their risk for COVID-19. At the same time, these practices faced unique challenges in adapting a traditionally hands-on model of care to accommodate restrictions on in-person contact. In this symposium, we present innovative research highlighting the challenges faced by HBPC practices and patients during spring 2020, as well as their rapid innovations and adaptations. First, Ritchie et al. provide national context with findings from a survey of U.S. home-based primary care practices that highlights the field’s most pressing challenges and successful strategies. Shifting to the initial epicenter of the pandemic in New York City, Reckrey et al. present a qualitative study of the perspectives of paid and unpaid caregivers of dementia patients served by an HBPC practice, while Franzosa et al. describe care disruptions among individuals with dementia who died during the initial surge, using a novel chart-based abstraction technique. Finally, two studies (Gorbenko et al. and Kalicki et al.) explore HBPC practices’ experience of rapidly transitioning to telehealth through qualitative interviews with NYC-based practices and a provider survey exploring telehealth adoption and readiness in the homebound population. Together, these studies yield important insights into the challenges of providing community-based care for at-risk populations during a pandemic, and practical strategies for home-based models of care moving forward.

